# The Protein Chaperone ClpX Targets Native and Non-native Aggregated Substrates for Remodeling, Disassembly, and Degradation with ClpP

**DOI:** 10.3389/fmolb.2017.00026

**Published:** 2017-05-04

**Authors:** Christopher J. LaBreck, Shannon May, Marissa G. Viola, Joseph Conti, Jodi L. Camberg

**Affiliations:** Department of Cell and Molecular Biology, University of Rhode IslandKingston, RI, USA

**Keywords:** disaggregation, proteolysis, unfoldase, ATPase, AAA+

## Abstract

ClpX is a member of the Clp/Hsp100 family of ATP-dependent chaperones and partners with ClpP, a compartmentalized protease, to degrade protein substrates bearing specific recognition signals. ClpX targets specific proteins for degradation directly or with substrate-specific adaptor proteins. Native substrates of ClpXP include proteins that form large oligomeric assemblies, such as MuA, FtsZ, and Dps in *Escherichia coli*. To remodel large oligomeric substrates, ClpX utilizes multivalent targeting strategies and discriminates between assembled and unassembled substrate conformations. Although ClpX and ClpP are known to associate with protein aggregates in *E. coli*, a potential role for ClpXP in disaggregation remains poorly characterized. Here, we discuss strategies utilized by ClpX to recognize native and non-native protein aggregates and the mechanisms by which ClpX alone, and with ClpP, remodels the conformations of various aggregates. We show that ClpX promotes the disassembly and reactivation of aggregated Gfp-ssrA through specific substrate remodeling. In the presence of ClpP, ClpX promotes disassembly and degradation of aggregated substrates bearing specific ClpX recognition signals, including heat-aggregated Gfp-ssrA, as well as polymeric and heat-aggregated FtsZ, which is a native ClpXP substrate in *E. coli*. Finally, we show that ClpX is present in insoluble aggregates and prevents the accumulation of thermal FtsZ aggregates *in vivo*, suggesting that ClpXP participates in the management of aggregates bearing ClpX recognition signals.

## Introduction

Maintaining cellular proteostasis relies on chaperone pathways that promote native protein folding. Typical strategies include targeting misfolded, unfolded, and aggregated polypeptides for reactivation or degradation (Bukau and Horwich, 1998; Wickner et al., 1999; Stoecklin and Bukau, 2013). Misfolded proteins are generated during polypeptide elongation and as a complication of environmental stress (Powers and Balch, 2013). The challenges imposed on chaperone systems by proteotoxic stress are especially relevant in pathogenic organisms like *E. coli*, which experience extreme fluctuations in environmental conditions leading to accumulation of protein aggregates and subsequent proteotoxicity (Mogk et al., 2011). Protein quality control systems reactivate, degrade and remove damaged and aggregated proteins. Under thermal stress in *E. coli*, the heat shock response provides a cellular defense mechanism and upregulates heat shock protein and chaperone levels to restore proteostasis (Mogk et al., 2011).

In addition to preventing protein aggregation, chaperone proteins mediate aggregate clearance through proteolysis of non-native proteins and aggregation reversal (Hartl et al., 2011; Mogk et al., 2011). Clearance of misfolded proteins in *E. coli* is carried out by AAA+ (ATPases Associated with diverse cellular Activities) proteins, which initiate substrate recognition, unfolding, and translocation into a proteolytic chamber (ClpP, HslV; Snider and Houry, 2008; Sauer and Baker, 2011). Several AAA+ proteins, such as Lon and FtsH, contain both AAA+ chaperone and proteolytic domains within a single protomer (Sauer and Baker, 2011). The chaperone-protease Lon recognizes exposed aromatic and hydrophobic residues, which may contribute to less stringent substrate selectivity and favor degradation of unfolded or misfolded proteins (Gur and Sauer, 2008).

The Clp ATPases of the AAA+ superfamily can be separated into two functional categories: degradation or disaggregation machines. Degradation machines, including ClpX, ClpA, and HslU form complexes with peptidases ClpP or HslV to remove misfolded proteins or specific substrates (Zolkiewski, 2006). Disaggregation machines, including Hsp104 and its bacterial homolog ClpB, disaggregate and reactivate aggregated proteins by an ATP-dependent mechanism and can function in cooperation with the Hsp70/DnaK system independent of protein degradation (Zolkiewski, 1999; Dougan et al., 2002; Doyle et al., 2007; Sweeny and Shorter, 2016). Through a collaborative mechanism, Hsp70, with Hsp40, binds first to a polypeptide segment of an aggregated protein and then the substrate is remodeled by Hsp104/ClpB (Zietkiewicz et al., 2004, 2006; Acebrón et al., 2009).

*E. coli* substrates that are degraded by ClpXP include a variety of cellular proteins, metabolic enzymes and several proteins capable of forming large conformational assemblies, including FtsZ, Dps, and MinD (Flynn et al., 2003; Stephani et al., 2003; Neher et al., 2006; Camberg et al., 2009, 2014; Conti et al., 2015). ClpXP can associate with cellular aggregates in *E. coli* and can promote removal of cellular inclusions, but direct protein disaggregation *in vitro* is not well characterized for ClpX (Vera et al., 2005; Winkler et al., 2010). An early study suggested that ClpX, in the absence of ClpP, could protect the lambda O phage protein from aggregation and resolubilize lambda O aggregates (Wawrzynow et al., 1995). In *Bacillus subtilis*, ClpX also localizes to protein aggregates, suggesting that it may be involved in protein disaggregation (Kruger et al., 2000; Kain et al., 2008; Kirstein et al., 2008; Simmons et al., 2008). ClpX and ClpX substrates are present in polar protein aggregates in *E. coli* under stress *in vivo*, suggesting that ClpX associates with aggregated proteins and participates in their removal (Kain et al., 2008; Maisonneuve et al., 2008; Simmons et al., 2008).

ClpXP comprises an asymmetric, hexameric ring of ClpX docked to two stacked heptameric rings of the ClpP serine protease (Wang et al., 1997; Glynn et al., 2009). Although ClpX has been shown to independently remodel substrates, such as MuA, in the presence of ClpP, hydrophobic “IGF” loops on the bottom surface of the ClpX hexamer contact hydrophobic pockets on the ClpP tetradecamer, allowing unfolded substrates to access the ClpP proteolytic chamber (Kim et al., 2001; Abdelhakim et al., 2010; Baker and Sauer, 2012). Nucleotide binding by ClpX protomers, in the cleft between the large and small AAA+ subdomains, regulate the position of the subdomains relative to each other; these conformational changes enable ClpX to couple substrate translocation to ATP hydrolysis (Glynn et al., 2009; Baker and Sauer, 2012). Substrates are then translocated into the ClpP chamber for degradation (Baker and Sauer, 2012).

Substrates bind to the ClpX N-domain and to residues in the ClpX central channel (pore-loops; Bolon et al., 2004; Park et al., 2007; Martin et al., 2008; Baker and Sauer, 2012). The N-domain of ClpX is separated from the AAA+ domain by a flexible linker and can dimerize independently. The N-domain is important for direct recognition of some substrates, including FtsZ and MuA, as well as adaptor proteins, but is not required for direct recognition of the ssrA-tag (Abdelhakim et al., 2008; Martin et al., 2008; Camberg et al., 2009; Baker and Sauer, 2012). Adaptor proteins, such as RssB or SspB, promote the interaction and engagement of specific substrates, such as RpoS or ssrA-tagged substrates, respectively (Sauer and Baker, 2011). The ssrA tag is an 11-residue degron appended to a nascent polypeptide when the ribosome stalls during protein synthesis, targeting the misfolded protein for subsequent degradation (Gottesman et al., 1998; Levchenko et al., 2000).

ClpXP is implicated in the degradation of diverse cellular substrates and more than 100 substrates have been reported (Flynn et al., 2003; Neher et al., 2006). Native substrates of ClpX contain recognition motifs at the N- or C-termini (Flynn et al., 2003). Notably, the essential cell division protein FtsZ in *E. coli* has two distinct ClpX motifs: one in the flexible linker region and one near the C-terminus (Camberg et al., 2014). FtsZ is a tubulin homolog that assembles into linear polymers *in vitro* and forms the septal ring critical for division *in vivo*, called the Z-ring (Erickson et al., 2010). ClpXP degrades ~15% of FtsZ proteins during the cell cycle in *E. coli* and is capable of degrading both monomers and polymers *in vitro* (Camberg et al., 2009). ClpXP degrades polymers more efficiently, which is consistent with a common strategy of multivalent recognition of substrates by AAA+ ATPases (Davis et al., 2009; Camberg et al., 2014; Ling et al., 2015). In addition to FtsZ, several other ClpXP substrates form large oligomeric structures, including the tetrameric phage protein MuA, the dodecameric bacterial protein Dps, and the bacterial cell division ATPase MinD (Stephani et al., 2003; Neher et al., 2006; Abdelhakim et al., 2010; Conti et al., 2015). Like FtsZ, alternate monomeric and oligomeric conformations of MuA are also differentially recognized by ClpX (Abdelhakim et al., 2008, 2010; Ling et al., 2015).

In this study, we use engineered and native substrates to investigate the role of ClpX and ClpXP in the disassembly and degradation of protein aggregates that bear specific ClpX recognition signals. We observed that ClpX, with and without ClpP, destabilizes Gfp-ssrA aggregates *in vitro*. The native ClpXP substrate FtsZ forms several discrete conformations, including linear ordered polymers and also heat-induced aggregates. Our results show that ClpXP disassembles both heat-induced and linear polymers containing FtsZ. Finally, we also demonstrate that thermal stress promotes aggregation of FtsZ, which is exacerbated in cells deleted for *clpX* or *clpP*. Together, these results show bona fide chaperone activity for ClpX *in vitro* and suggest that ClpX, with or without ClpP, may play a broader role in rescue and disassembly of protein aggregates.

## Materials and methods

### Bacterial strains and plasmids

*E. coli* strains and plasmids used in this study are described in Table 1. An expression plasmid encoding FtsZ(ΔC67) was constructed by introducing a TAA stop codon (at residue 317 of FtsZ) into pET-FtsZ by site-directed mutagenesis (Camberg et al., 2009).

**Table 1:** *****E. coli*** strains and plasmids used in this study**.

**Strain or plasmid**	**Genotype**	**Source, reference or Construction**
**STRAINS**		
BW25113	F^−^, DE(*araD-araB*)567, lacZ4787(del)(::rrnB-3), *LAM-, rph-1*, DE(*rhaD-rhaB*)*568, hsdR514*	Datsenko and Wanner, 2000
JW0429	F-, Δ*(araD-araB)567, ΔlacZ4787*(::rrnB-3), Δ*lon-725::kan, λ^−^, rph-1, Δ(rhaD-rhaB)568, hsdR514*	Baba et al., 2006
JW0428	F-, Δ*(araD-araB)567,ΔlacZ4787*(::rrnB-3), Δ*clpX724::kan, λ^−^, rph-1, Δ(rhaD-rhaB)568, hsdR514*	Baba et al., 2006
JW0427	F-, Δ*(araD-araB)567, ΔlacZ4787*(::rrnB-3), Δ*clpP723::kan, λ^−^, rph-1, Δ(rhaD-rhaB)568, hsdR514*	Baba et al., 2006
JW2573	F-, Δ*(araD-araB)567, ΔlacZ4787*(::rrnB-3), Δ*clpB757::kan, λ^−^, rph-1, Δ(rhaD-rhaB)568, hsdR514*	Baba et al., 2006
JW0866	F-, Δ*(araD-araB)567, ΔlacZ4787*(::rrnB-3), Δ*clpA783::kan, λ^−^, rph-1, Δ(rhaD-rhaB)568, hsdR514*	Baba et al., 2006
JW3902	F-, Δ*(araD-araB)567, ΔlacZ4787*(::rrnB-3), Δ*hslU790::kan, λ^−^, rph-1, Δ(rhaD-rhaB)568, hsdR514*	Baba et al., 2006
JW3903	F-, Δ*(araD-araB)567, ΔlacZ4787*(::rrnB-3), Δ*hslV720::kan, λ^−^, rph-1, Δ(rhaD-rhaB)568, hsdR514*	Baba et al., 2006
JW0013	F-, Δ*(araD-araB)567, ΔlacZ4787*(::rrnB-3), Δ*dnaK734::kan, λ^−^, rph-1, Δ(rhaD-rhaB)568, hsdR514*	Baba et al., 2006
JW0462	F-, Δ*(araD-araB)567, ΔlacZ4787*(::rrnB-3), Δ*htpG757::kan, λ^−^, rph-1, Δ(rhaD-rhaB)568, hsdR514*	Baba et al., 2006
JC0259	MG1655 Δ*clpX::kan*	Camberg et al., 2011
**PLASMIDS**		
pET-ClpX	*kan*	Camberg et al., 2009
pET-ClpP	*kan*	Maurizi et al., 1994
pET-FtsZ	*kan*	Camberg et al., 2009
pET-FtsZ(ΔC67)	*kan*	This study
pET-H_6_-Gfp(uv)	*kan*	This study
pGfp-ssrA	*amp*	Singh et al., 2000
pClpX(E185Q)	*amp*	Camberg et al., 2011

### Expression and purification of proteins

Gfp-ssrA was purified as previously described (Yakhnin et al., 1998). ClpX, ClpP, FtsZ, and FtsZ(ΔC67) were each overexpressed in *E. coli* BL21 (λDE3) and purified as described (Maurizi et al., 1994; Grimaud et al., 1998; Camberg et al., 2009, 2014). ClpX(E185Q) was purified as described for wild type ClpX, except the expression strain, *E. coli* MG1655 Δ*clpX* carrying plasmid pClpX(E185Q), was induced with 1% arabinose (Table 1; Camberg et al., 2011). Gfp(uv) containing an N-terminal histidine tag was overexpressed in *E. coli* BL21 (λDE3) and grown to an OD_600_ of 1.0 and induced for 3 h at 30°C. Cells were lysed by French press in purification lysis buffer (20 mM HEPES, pH 7.5, 5 mM MgCl_2_, 50 mM KCl, and 10% glycerol). Soluble extracts were bound to TALON metal affinity resin (GE Healthcare), eluted with an imidazole gradient, and imidazole was removed by buffer exchange. Protein concentrations are reported as FtsZ monomers, ClpX hexamers, ClpP tetradecamers, and Gfp or Gfp-tagged monomers. For polymerization assays, FtsZ was labeled with Alexa Fluor 488 and active protein (FL-FtsZ) was collected after cycles of polymerization and depolymerization as described (González et al., 2003; Camberg et al., 2014).

### Dynamic light scattering

Dynamic light scattering (DLS) measurements were made using a Zetasizer Nano ZS (Malvern Instruments). To determine size distribution, FtsZ (5 μM), aggFtsZ (5 μM), Gfp-ssrA (1.5 μM), and aggGfp-ssrA (1.5 μM) in reaction buffer (50 mM HEPES, pH 7.5, 100 mM KCl and 10 mM MgCl_2_) were added to polystyrene cuvettes and scanned at 23°C with a detector angle of 173° and a 4 mW, 633 nm He–Ne laser. The reported intensity-weighted hydrodynamic diameters are based on 15 scans.

### Heat denaturation, aggregation, disassembly, and reactivation of aggregated substrates

To heat-inactivate Gfp substrates, Gfp-ssrA (1.5 μM) or Gfp(uv) (1.5 μM) was added, where indicated, to buffer containing HEPES (50 mM, pH 7.5), KCl (100 mM), MgCl_2_ (10 mM), glycerol (10%) and dithiothreitol (DTT) (2 mM) in a volume of 800 μl and incubated at 85°C for 15 min. Immediately following heat-treatment, the denatured substrate was placed on ice for 2 min and added to a reaction (50 μl) containing ClpX, (0.3 μM), ClpX (E185Q) (0.3 μM), ClpP (0.3 μM), ATP (4 mM), ATPγS (1 mM), or ADP (2 mM), where indicated. Samples containing ATP were supplemented with an ATP-regenerating system containing phosphocreatine (5 mg ml^−1^) and creatine kinase (CK) (60 μg ml^−1^). Fluorescence recovery was monitored by measuring fluorescence in a Cary Eclipse fluorometer with excitation and emission wavelengths set at 395 nm and 510 nm, respectively. Readings were corrected for background signal by subtracting the fluorescence of buffer. Rates were calculated by fitting to a one-phase association model in GraphPad Prism (version 6.0b). Disaggregation was monitored by 90°-angle light scatter with excitation and emission wavelengths set to 550 nm. Readings were corrected for background signal by subtracting the scatter of the buffer and then plotted as percent of the initial turbidity. Heat-induced aggregation of Gfp-ssrA with time was monitored by 90°-angle light scatter with the temperature of the cuvette holder set to 80°C using a circulating water bath.

To inactivate native FtsZ substrates, FtsZ and FtsZ(ΔC67) (5 μM) were heated for 15 min in reaction buffer (20 mM HEPES, pH 7.5, 100 mM KCl, 10 mM MgCl_2_) in a volume of 120 μl at 65°C, then cooled on ice for 40 s, and held at 23°C until addition to reactions (60 μl volume) containing ClpX (0.5 μM or 1 μM), ClpX(E185Q) (0.5 μM), ClpP (1 μM), ATP (4 mM) and an ATP-regenerating system (phosphocreatine at 5 mg ml^−1^ and creatine kinase at 60 μg ml^−1^), where indicated. Disaggregation was monitored by 90°-angle light scatter with excitation and emission wavelengths set to 450 nm. Readings were corrected for background signal by subtracting the scatter of the buffer and then plotted as percent of the initial turbidity. Heat-induced aggregation of FtsZ with time was monitored by 90°-angle light scatter with the temperature of the cuvette holder set to 65°C using a circulating water bath.

### Polymerization and GTP hydrolysis assays

FL-FtsZ was incubated with the GTP analog GMPCPP (0.5 mM) in the presence of increasing concentrations of ClpX and ClpP (0, 0.25, 0.5, or 1 μM) as indicated and in the presence of phosphocreatine at 5 mg ml^−1^ and creatine kinase at 60 μg ml^−1^. Samples were incubated for 3 min in buffer containing MES (50 mM, pH 6.5), KCl (100 mM) and MgCl_2_ (10 mM) at 23°C, then centrifuged at 129,000 × g in a Beckman TLA 120.1 rotor for 30 min. Pellets were resuspended in 0.2 M NaCl with 0.01% Triton X-100 (100 μl) and the fluorescence associated with FL-FtsZ for supernatants and pellets was measured using a Cary Eclipse spectrophotometer. GTP hydrolysis rates for FtsZ and FtsZ(ΔC67) were measured before and after aggregation using the Biomol Green (Enzo Life Sciences) detection reagent as described (Camberg et al., 2014).

### Heat shock of wild type and deletion strains

*E. coli* wild type and deletion strains were grown overnight, diluted 1:100 in fresh Lennox broth the next day and grown at 30°C to an OD of 0.4. All strains were incubated in a water bath at 50°C for 1 h, followed by recovery at 30°C for 35 min. Cells were harvested by centrifugation and lysed with Bacterial Protein Extraction Reagent (B-PER) (ThermoFisher Scientific) (2 ml) and lysozyme (25 μg ml^−1^). Insoluble fractions were collected by centrifugation at 15,000 × g for 5 min at 4°C, resuspended in lithium dodecyl sulfate sample buffer and analyzed by reducing SDS-PAGE. Total proteins were transferred to a nitrocellulose membrane and visualized by Ponceau (Fisher Scientific) staining and membranes were immunoblotted using antibodies to ClpX and FtsZ (Camberg et al., 2009, 2011). Band intensities were analyzed by densitometry (NIH ImageJ), normalized to the intensity of the average of the “no heat” sample, and evaluated for significance by the Mann-Whitney test. Where indicated, to test a mild heat shock condition, cells were incubated in a water bath at 42°C for 30 min, followed by recovery at 30°C for 35 min, and analyzed as described.

## Results

### ClpXP degrades aggregates *In vitro*

**Figure 1:**
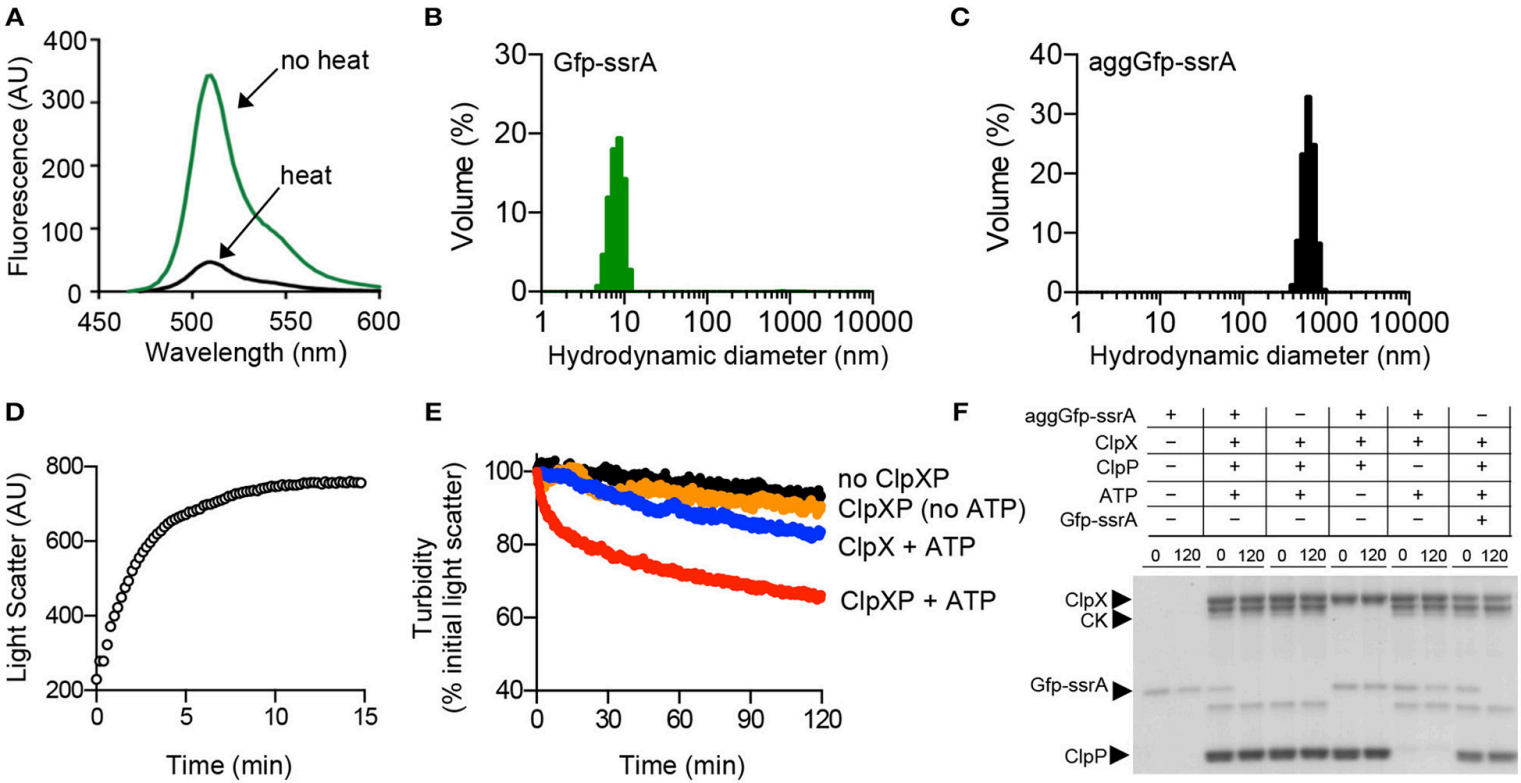
**Disaggregation and degradation of aggregated Gfp-ssrA by ClpXP. (A)** The fluorescence emission spectra (450–600 nm) of Gfp-ssrA (1.0 μM) (green) and heat-treated Gfp-ssrA (1.0 μM) (black) (85°C for 15 min) were measured using an excitation wavelength of 395 nm. Plotted curves are representative of three traces. **(B)** DLS was performed for Gfp-ssrA (1.0 μM) (green) as described to determine particle size (nm) distribution. **(C)** DLS was performed for heat-treated Gfp-ssrA (aggGfp-ssrA) (1.0 μM) (black) as described to determine particle size (nm) distribution. **(D)** Aggregation by 90°–angle light scatter was measured for Gfp-ssrA (1.5 μM) (open circles) in a cuvette attached to a circulating water bath held at 80°C. Light scattering was monitored for 15 min. **(E)** Disaggregation of aggGfp-ssrA (1 μM) was monitored by 90°–angle light scatter as described in Materials and Methods. Disaggregation reactions contained aggGfp-ssrA (1 μM) (black circles), ClpX (0.5 μM) and ATP (blue circles), ClpX (0.5 μM), and ClpP (0.6 μM) (gold circles), ClpX (0.5 μM), ClpP (0.6 μM), and ATP (4 mM) (red circles), and a regenerating system, where indicated. Light scattering was monitored for 120 min. Curves shown are representative of at least three replicates. **(F)** Degradation of Gfp-ssrA and aggGfp-ssrA was monitored as described in Materials and Methods in reactions containing Gfp-ssrA (1 μM) or aggGfp-ssrA (1 μM), where indicated, and ClpX (0.5 μM), ClpP (0.6 μM), ATP (4 mM), and a regenerating system, where, indicated. Reactions were incubated at 23°C for 120 min and samples were analyzed by SDS-PAGE and Coomassie stain.

To determine if ClpX can remodel protein substrates from the aggregated state, we used the fusion protein, Gfp-ssrA, which forms aggregates upon heat treatment (Zietkiewicz et al., 2004, 2006). Gfp-ssrA is rapidly degraded by ClpXP and has been extensively studied to understand substrate targeting by ClpXP. The Gfp moiety is widely used in protein disaggregation assays because it forms non-fluorescent aggregates when heated, but is disaggregated and reactivated by several chaperone systems (Zietkiewicz et al., 2004, 2006). Therefore, we heated Gfp-ssrA at 85°C for 15 min to induce aggregation (aggGfp-ssrA), resulting in an 86% loss of fluorescence emitted (Figure 1A). Next, to measure the distribution of aggregates by size after heating, we performed dynamic light scattering (DLS) of untreated and heat-denatured Gfp-ssrA. We observed that without heating, the particle sizes are uniform with an average hydrodynamic diameter of 8–10 nm (Figure 1B). After heating, aggregates are ~500–600 nm, and there is a narrow distribution of particle sizes and no small particles (i.e., <100 nm; Figure 1C). Upon heat-treatment, aggregation of Gfp-ssrA (1.5 μM) occurs rapidly and plateaus by 10 min by 90°-angle light scattering (Figure 1D). The heat inactivation is irreversible since incubation of aggregated Gfp-ssrA (aggGfp-ssrA) alone does not lead to appreciable fluorescence reactivation, which is consistent with previous reports using Gfp (Figure S1; Zietkiewicz et al., 2004). To determine if ClpXP can bind to aggregates and degrade them, we incubated aggGfp-ssrA with ClpXP and monitored turbidity by 90°-angle light scattering. Incubation of aggGfp-ssrA with ClpXP led to a 35% loss of turbidity in 2 h (Figure 1E). However, when ClpXP was omitted from the reaction, there was very little change in turbidity over time (5% loss in 2 h; Figure 1E). This suggests that ClpXP targets aggregated substrates for degradation. To determine if degradation is required to reduce turbidity, we omitted ClpP and observed that ClpX is capable of reducing sample turbidity by 15% in 2 h (Figure 1E). Finally, when ATP was omitted from the reaction containing ClpXP, we observed a <10% reduction in the turbidity of the reaction (Figure 1E). To confirm that ClpXP degrades aggGfp-ssrA, we incubated aggGfp-ssrA with combinations of ClpX, ClpP, and ATP, and sampled degradation reactions after 2 h. We observed that in the presence of ClpXP, aggGfp-ssrA is degraded, but not when ClpP or ATP was omitted (Figure 1F). Together, these results demonstrate that ClpXP targets aggregates for ATP-dependent degradation and that ClpX is also capable of promoting disassembly in the absence of ClpP.

**Figure 2:**
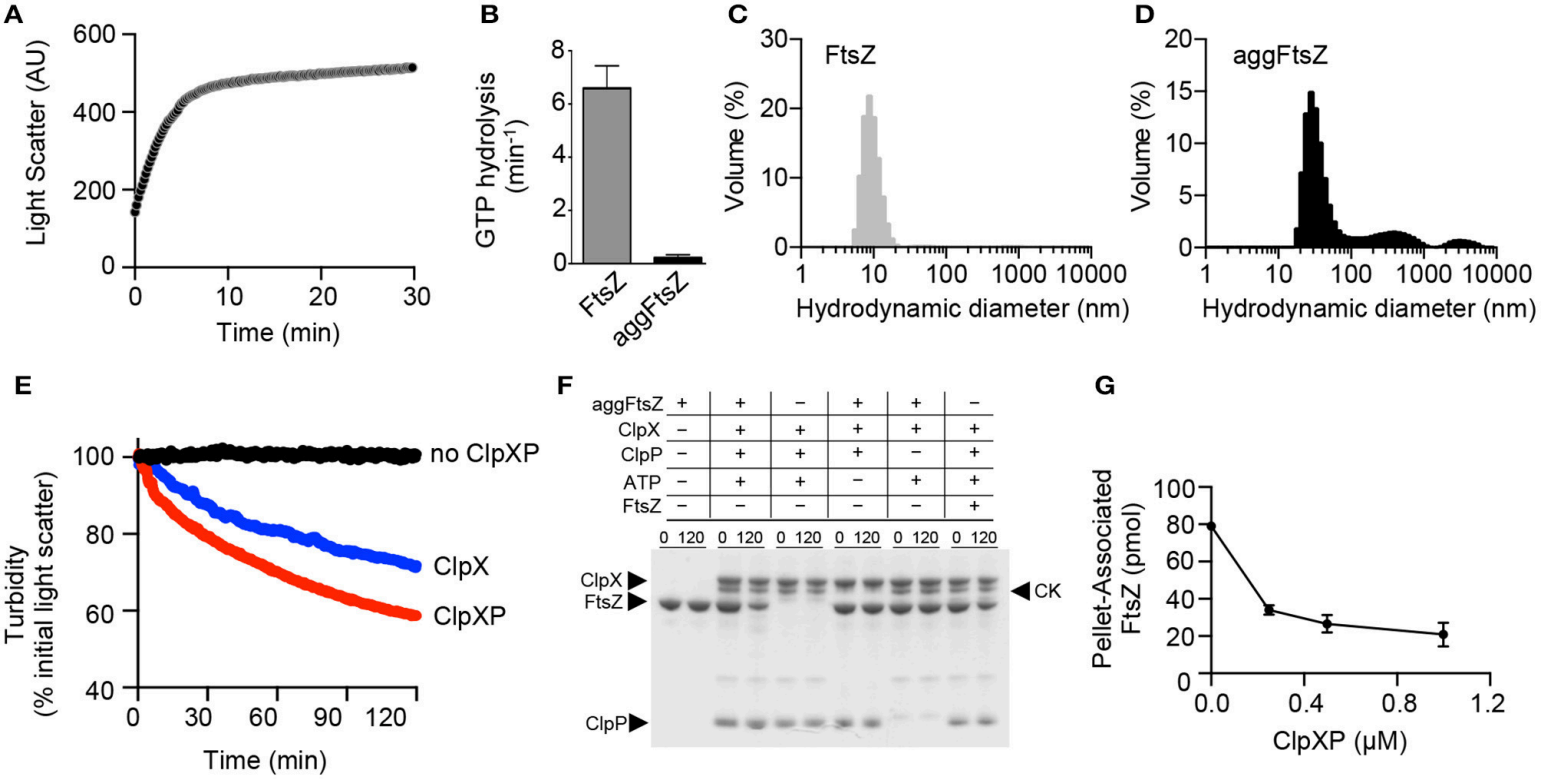
**Aggregation and disaggregation of native ClpXP substrate FtsZ. (A)** Aggregation by 90°–angle light scatter was measured for FtsZ (5 μM) (black circles) in a cuvette attached to a circulating water bath at 65°C for 30 min as described in Materials and Methods. The curve shown is representative of at least three replicates. **(B)** Rates of GTP hydrolysis were measured for FtsZ (5 μM) (gray) and aggFtsZ (5 μM) (black) with GTP (1 mM) for 15 min at 30°C, as described in Materials and Methods. The average rate was determined from at least four replicates. **(C)** DLS was performed for FtsZ (5 μM) (gray) as described to determine particle size (nm) distribution. **(D)** DLS was performed for aggFtsZ (5 μM) (black) as described to determine particle size (nm) distribution. **(E)** Disaggregation of aggFtsZ (5 μM) was monitored by 90°-angle light scatter as described in Materials and Methods. Disaggregation reactions contained aggFtsZ (5 μM) (black circles) or aggFtsZ (5 μM) and ClpX (1 μM) (blue circles), or aggFtsZ (5 μM), ClpX (1 μM), and ClpP (1 μM) (red circles), with ATP (4 mM) and a regenerating system. Light scattering was monitored for 120 min. The curves shown are representative of at least three replicates. **(F)** Degradation was monitored for FtsZ and aggFtsZ as described in Materials and Methods in reactions containing FtsZ (6 μM), aggFtsZ (6 μM), ClpX (0.5 μM), ClpP (0.5 μM), ATP (4 mM) and a regenerating system, where indicated. For degradation of FtsZ, GMPCPP (0.5 mM) was included to promote the assembly of stable polymers. Degradation reactions were incubated at 23°C for 120 min. To detect protein loss due to degradation, samples from 0 and 120 min were analyzed by SDS-PAGE to solubilize any remaining aggregates. **(G)** Degradation was monitored for FL-FtsZ (125 pmol) incubated in the presence of GMPCPP (0.5 mM) for 3 min, then ATP (4 mM), a regenerating system, and increasing concentrations of ClpXP (0, 0.25, 0.5, and 1 μM as shown) were added and reactions were incubated for an additional 30 min at 23°C. Reactions were centrifuged at 129,000 × g for 30 min at 23°C. Pellet-associated FtsZ was quantified by fluorescence, and each data point is an average of at least three replicates.

FtsZ is a well-characterized ClpXP substrate that is essential for cell division and forms linear polymers *in vitro* in the presence of GTP (Erickson et al., 2010). We previously showed that ClpXP binds to GTP-stimulated FtsZ polymers and promotes FtsZ degradation (Camberg et al., 2009). ClpXP also recognizes and degrades non-polymerized FtsZ, but less efficiently than polymerized FtsZ (Camberg et al., 2009). *In vitro*, FtsZ rapidly aggregates when heated at 65 °C and this aggregation is associated with an increase in overall light scatter and a 97% loss of GTPase activity (Figures 2A,B). FtsZ, which purifies as a mixture of monomers (40.4 kDa) and dimers (80.8 kDa), has an average hydrodynamic diameter of 10–15 nm by DLS (Figure 2C). Heat treatment of FtsZ (5 μM) at 65°C produces several particle sizes, including small (30–40 nm) and large aggregates (>300 nm; Figure 2D). To determine if ClpXP reduces the turbidity associated with aggregated FtsZ (aggFtsZ), we incubated aggFtsZ with ClpXP and ATP and observed a 40% loss of turbidity after incubation with ClpXP for 2 h (Figure 2E). However, in the absence of ClpXP, the light scatter signal remained stable for aggFtsZ (Figure 2E). Incubation of ClpX with aggFtsZ also resulted in a 25% loss in light scatter, suggesting that ClpX also promotes disassembly of aggregates similar to what we observed for aggGfp-ssrA (Figures 1E, 2E).

Next, to confirm that aggFtsZ is degraded by ClpXP, we assembled reactions containing combinations of aggFtsZ, ClpX, ClpP, and ATP and sampled these reactions at 0 and 120 min for analysis by SDS-PAGE. We observed that in the presence of ClpXP and ATP, 50% of the total aggFtsZ in the reaction is lost to degradation after 120 min (Figure 2F). Omission of either ClpP or ATP from the reaction prevents loss of aggFtsZ (Figure 2F). These results indicate that ClpXP degrades aggFtsZ. Furthermore, the amount of aggFtsZ after incubation with ClpX is unchanged despite the decrease in light scatter detected, suggesting that ClpX can disaggregate aggFtsZ (Figures 2E,F).

In addition to forming aggregates upon heating, FtsZ also assembles into a linear head-to-tail polymer, which is a native, ordered aggregate, and distinct from the disordered aggregates which are induced by heating (aggFtsZ). We compared the loss of aggFtsZ by ClpXP to a similar reaction monitoring loss of native polymerized FtsZ, which is a known substrate of ClpXP. Like aggFtsZ, we also observed a ~50% loss of polymeric FtsZ, stabilized by the GTP analog GMPCPP, after 120 min in reactions containing ClpXP and ATP (Figure 2F). GMPCPP promotes the assembly of stable polymers that are far less dynamic than polymers assembled with GTP (Lu et al., 2000). To test if ClpXP disassembles GMPCPP-stabilized FtsZ polymers, we incubated pre-assembled polymers with ClpXP and ATP. Then, we collected polymers by high-speed centrifugation. In these assays, we used active fluorescent FtsZ, labeled with Alexa fluor 488 (FL-FtsZ), to quantify the amount of polymerized FtsZ in the pellet fraction and soluble FtsZ in the supernatant. We observed that after incubation of GMPCPP-stabilized FtsZ polymers with increasing concentrations of ClpXP (0–1 μM), few FtsZ polymers were recovered in the pellet fractions containing ClpXP (26% of the total FtsZ was recovered in the reaction containing 1 μM ClpXP), indicating that ClpXP is highly effective at promoting the disassembly of GMPCPP-stabilized FtsZ polymers (Figure 2G).

### ClpX reactivates heat-aggregated Gfp-ssrA

**Figure 3:**
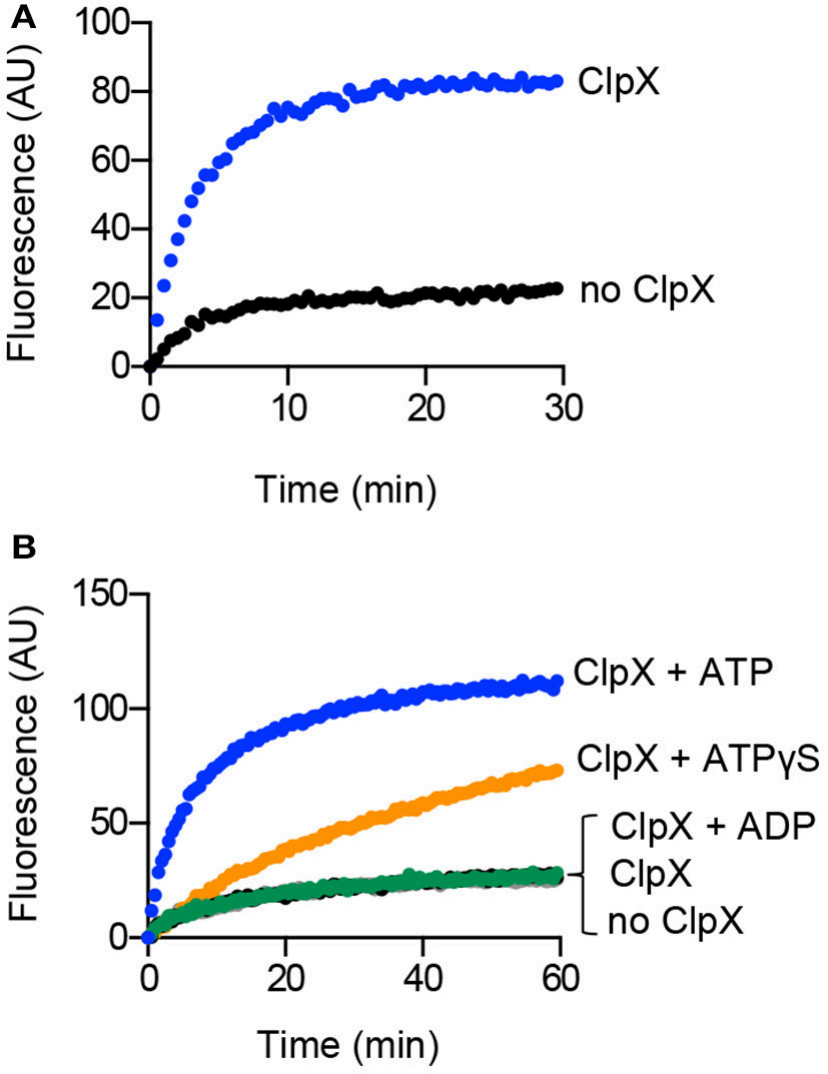
**Reactivation of aggregated Gfp-ssrA in the presence of ClpX. (A)** Reactivation of aggGfp-ssrA (1.0 μM) was monitored as described in Materials and Methods in the absence (black circles) and presence (blue circles) of ClpX (0.3 μM), ATP (4 mM), and a regenerating system. Fluorescence emission (AU) was monitored for 30 min. The curves shown are representative of at least three replicates. **(B)** Reactivation of aggGfp-ssrA (1.0 μM) was monitored in the absence (black circles) or presence of ClpX (0.3 μM), ATP (4 mM) and a regenerating system (blue circles), ATPγS (2 mM) (orange circles), ADP (2 mM) (green circles), or no nucleotide (gray circles), where indicated. Fluorescence emission (AU) was monitored for 60 min. The curves shown are representative of at least three replicates.

Incubation of ClpX with aggGfp-ssrA resulted in loss of turbidity, suggesting that ClpX may function independently of ClpP to reactivate substrates (Figure 1E). Reactivation of misfolded proteins may occur through binding and stabilization of intermediates enabling proteins to adopt the native folded conformation, or through ATP-dependent chaperone-assisted unfolding. To determine if ClpX, which recognizes the ssrA amino acid sequence, is able to reactivate aggGfp-ssrA, we monitored fluorescence of aggGfp-ssrA in the presence and absence of ClpX and ATP. AggGfp-ssrA regains very little fluorescence alone, ~20 units, which is 8% of the initial fluorescence lost upon heating; however, in the presence of ClpX, fluorescence recovers rapidly in the first 10 min of the reaction and then plateaus, regaining ~85 units, which is 27% of the initial fluorescence lost upon heating (Figure 3A).

ClpX catalyzes ATP-dependent unfolding of substrates (Kim et al., 2000; Singh et al., 2000). To determine if ATP is essential for reactivation, we incubated aggGfp-ssrA with ClpX under various nucleotide conditions including with ATP, the ATP analog ATPγS, ADP and omission of nucleotide. We observed an 82% slower rate of fluorescence reactivation when ClpX and aggGfp-ssrA were incubated with ATPγS than with ATP (0.02 and 0.11 AU min^−1^, respectively), and no recovery over background with ADP or without nucleotide (Figure 3B). Reactivation by ClpX and ATP is prevented in the presence of ClpP, and the residual fluorescence after heat treatment is lost upon degradation (Figure S2). Together, these results indicate that ClpX requires ATP to reactivate Gfp-ssrA and, surprisingly, that ATPγS is also capable of promoting reactivation, although at a much slower rate than ATP (Figure 3B).

### Reactivation and disaggregation by ClpX requires a specific recognition sequence

**Figure 4:**
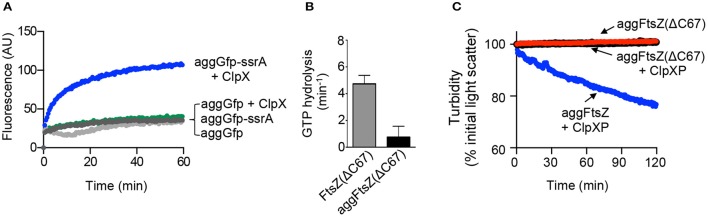
**Aggregation and disaggregation of ClpXP substrates with and without recognition motifs. (A)** Reactivation of aggGfp-ssrA (1.0 μM) alone (dark gray circles) or in the presence of ClpX (0.3 μM) (blue circles), and reactivation of aggGfp(uv) alone (1.0 μM) (light gray circles) or in the presence of ClpX (0.3 μM) (green circles), where indicated, was monitored with ATP (4 mM) and a regenerating system as described in Materials and Methods. Fluorescence emission (AU) was monitored for 60 min. The plotted curves are representative of at least three replicates. **(B)** Rates of GTP hydrolysis were determined as described in Materials and Methods for FtsZ(ΔC67) (5 μM) (gray) and aggFtsZ(ΔC67) (5 μM) (black), where indicated, incubated with GTP (1 mM) for 15 min at 30°C. The average rate was determined from at least four replicates. **(C)** Disaggregation was monitored by 90°-angle light scatter for aggFtsZ(ΔC67) (5 μM) alone (black), aggFtsZ(ΔC67) (5 μM) in the presence of ClpXP (0.5 μM), ATP (4 mM) and a regenerating system (red), or aggFtsZ (5 μM) in the presence of ClpXP (0.5 μM), ATP (4 mM) and a regenerating system (blue) where indicated as described in Materials and Methods. Light scattering was monitored for 120 min. The curves shown are representative of at least three replicates.

Next, we determined if a ClpX recognition motif is important for efficient recognition of aggregated substrates by ClpX. We compared reactivation of aggGfp-ssrA with heat-aggregated Gfp (aggGfp) without an ssrA tag. We observed that after incubation with ClpX and ATP for 60 min, ~30 units of fluorescence were recovered, which is 8% of the initial pre-heat fluorescence, indicating that aggGfp is a poor substrate for reactivation by ClpX (Figure 4A). In contrast, aggGfp-ssrA recovered 33% (>100 units) of the initial pre-heat fluorescence after incubation with ClpX (Figure 4A).

Two regions of FtsZ are important for promoting degradation of *E. coli* FtsZ by ClpXP, one in the unstructured linker region (amino acids 352–358) and one near the C-terminus (residues 379 through 383; Camberg et al., 2014). Using a truncated FtsZ mutant protein, FtsZ(ΔC67), which is deleted for 67 C-terminal amino acid residues, including both regions involved in ClpX recognition, we tested if ClpXP reduces the light scatter in reactions containing heat-aggregated FtsZ(ΔC67) [aggFtsZ(ΔC67)]. We heated FtsZ(ΔC67) at 65°C for 15 min, the condition that promotes aggregation of full length FtsZ, and confirmed that heat treatment resulted in an 84% loss of GTP hydrolysis activity and an increase in light scatter, which is stable over time (Figures 4B,C). In the presence of ClpXP, we observed no decrease in light scatter for aggFtsZ(ΔC67) after incubation for 120 min (Figure 4C), which is expected since FtsZ(ΔC67) is a poor substrate for ClpXP degradation (Figure S3). Together, these results demonstrate that for ClpX to recognize aggregates and promote disaggregation, disassembly and/or reactivation, a ClpX recognition motif is required.

### Impaired reactivation by ClpX(E185Q)

**Figure 5:**
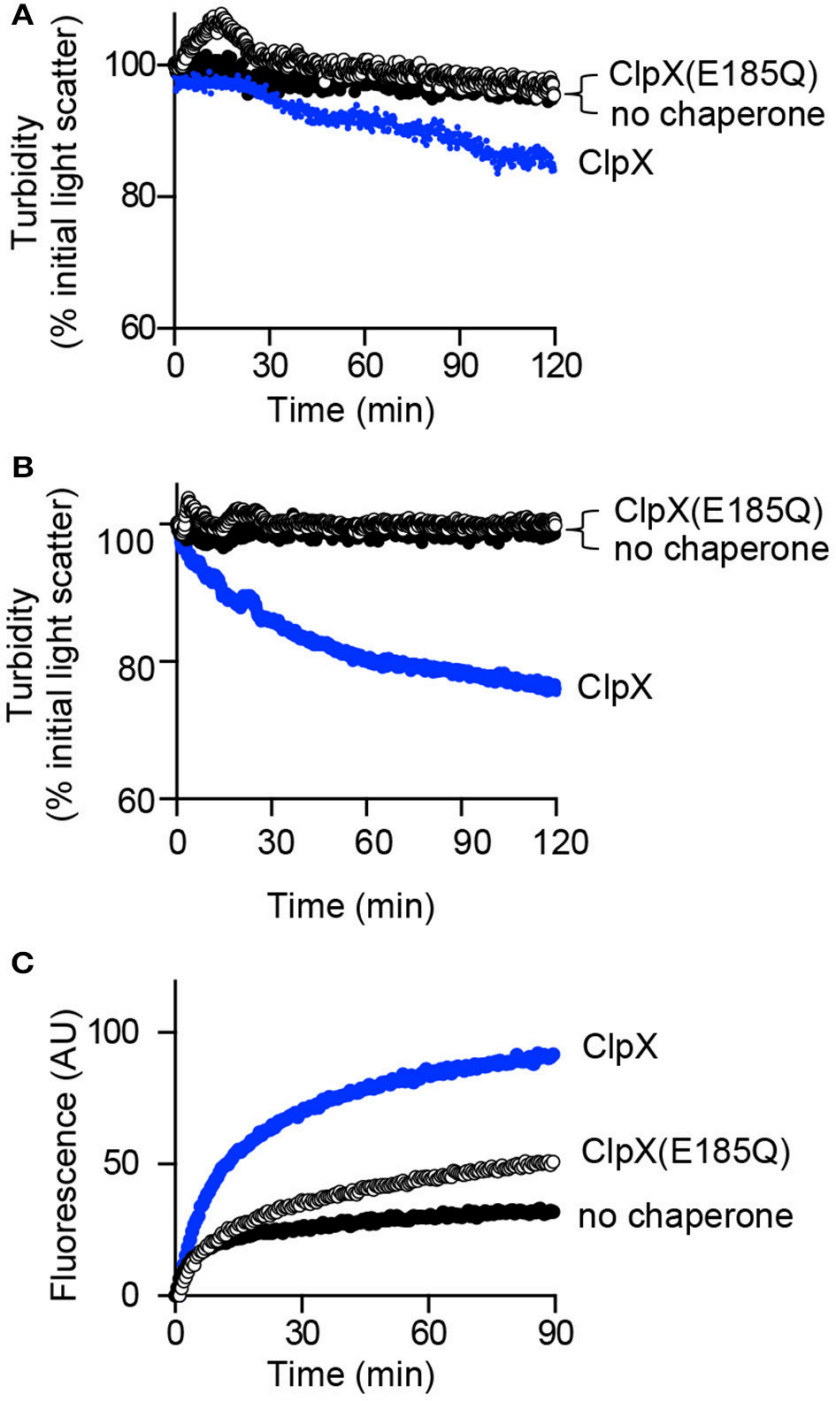
**Disaggregation and reactivation of ClpX substrates in the presence of ClpX(E185Q). (A)** Disaggregation was monitored by 90°-angle light scatter, as described in Materials and Methods for aggGfp-ssrA (1.0 μM) alone (black circles) or in the presence of ClpX (0.5 μM) (blue circles) or ClpX (E185Q) (0.5 μM) (open circles), where indicated, with ATP (4 mM), and a regenerating system. Light scattering was monitored for 120 min. The curves shown are representative of at least three replicates. **(B)** Disaggregation was monitored by 90°-angle light scatter for aggFtsZ (5 μM), ClpX (0.5 μM), or ClpX (E185Q) (0.5 μM) where indicated, ATP (4 mM), and a regenerating system for 120 min as described in Materials and Methods. The curves shown are representative of at least three replicates. **(C)** Reactivation was monitored as described in Materials and Methods for aggGfp-ssrA (1.0 μM) alone (black circles) or in the presence of ClpX (0.3 μM) (blue circles) or ClpX (E185Q) (0.3 μM) (open circles), with ATP (4 mM) and a regenerating system, where indicated. Fluorescence emission (AU) was monitored for 90 min. The curves shown are representative of at least three replicates.

ATP is required for reactivation of aggGfp-ssrA, however, it is unknown if this event requires ATP-hydrolysis and substrate unfolding. Therefore, we used the ClpX mutant protein ClpX(E185Q), which has a mutation in the Walker B motif and is defective for ATP-hydrolysis, but interacts with substrates (Hersch et al., 2005; Camberg et al., 2014). We observed that ClpX(E185Q) is defective for disaggregation of aggGfp-ssrA by monitoring turbidity by 90°-angle light scatter of reactions containing aggGfp-ssrA, ClpX(E185Q) and ATP (Figure 5A). We also tested if aggFtsZ is disassembled by ClpX(E185Q), and observed no reduction in light scatter in reactions containing aggFtsZ, ClpX(E185Q) and ATP after 120 min compared to ClpX (Figure 5B). Finally, we tested if reactivation of aggGfp-ssrA requires ATP hydrolysis using ClpX(E185Q) instead of ClpX. We observed that ClpX(E185Q) promotes a small amount of reactivation of aggGfp-ssrA and restores fluorescence, but to a much lesser extent than the level observed for wild type ClpX (Figure 5C). These results suggest that ATP hydrolysis by ClpX is required to promote efficient reactivation of aggGfp-ssrA and disassembly of large complexes containing aggFtsZ or aggGfp-ssrA (Figures 5A–C).

### ClpXP prevents accumulation of FtsZ aggregates *In vivo* under extreme thermal stress

**Figure 6:**
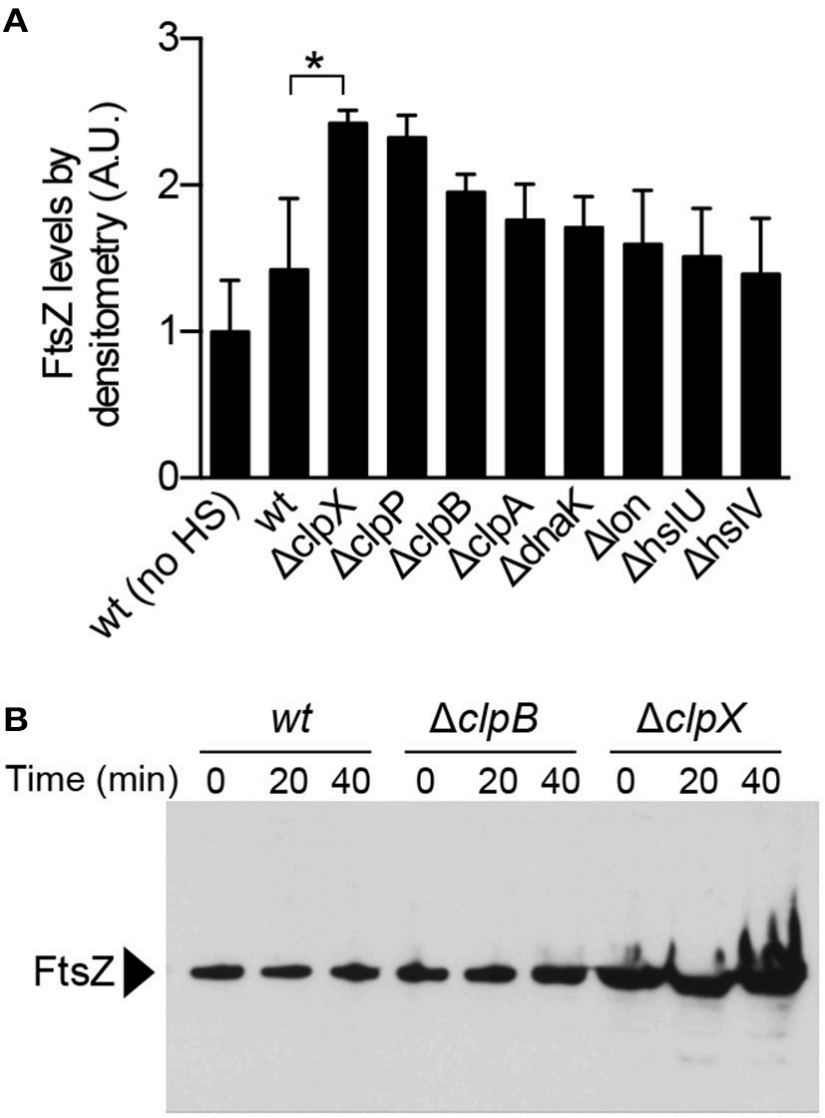
**FtsZ aggregation in deletion strains after heat shock. (A)** FtsZ levels were compared in insoluble cell extracts prepared from single gene deletion strains (Table 1) after heat shock at 50°C for 1 h and recovery (30°C) as described in Materials and Methods. Cells were collected and insoluble protein extracts were analyzed by immunoblotting using anti-FtsZ antibodies. Relative FtsZ levels were quantified by densitometry from four independent experiments. Where indicated, “^*^” represents a *p*-value of 0.03. **(B)** Insoluble FtsZ levels were monitored during the 30°C recovery period (0, 20, and 40 min) after heat shock at 50°C for 60 min in wild type, Δ*clpB* and Δ*clpX* deletion strains.

ClpX and ClpP were previously reported to localize to protein aggregates in *E. coli*, suggesting that ClpXP may target aggregates *in vivo* for direct degradation (Winkler et al., 2010). We used the native ClpXP substrate FtsZ, which aggregates upon heat treatment, to determine if ClpX and/or ClpXP modulates FtsZ aggregate accumulation after thermal stress by comparing the levels of FtsZ present in insoluble cell fractions (Figures 2A, 6A). Wild type cells and cells deleted for *clpX, clpP, clpB, clpA, dnaK, lon, hslU*, and *hslV* were exposed to heat shock and insoluble protein fractions were collected and analyzed by immunoblot. We observed that FtsZ was present in the insoluble fraction of wild type cells (BW25113), and this amount was 42% higher in cells exposed to heat shock at 50°C (Figure 6A and Figure S4A). However, FtsZ levels were even higher in the insoluble fractions of Δ*clpX* and Δ*clpP* strains compared to the parental strain (2.4-fold and 2.3-fold, respectively), although the amount of total protein was similar to the wild type strain exposed to heat shock (Figure S4B). We detected less protein overall in the Δ*dnaK* strain after recovery, but this strain also had poor viability after heat shock and recovery (Figure S4C). In addition, we also detected ClpX in the insoluble fraction in all strains except the *clpX* deletion strain (Figure S4A). Next, we conducted a mild heat shock, 42°C for 30 min, followed by recovery, and observed that deletion of *clpB* had a larger effect on the accumulation of insoluble FtsZ than deletion of *clpX* (Figure S4D). To determine the relative contributions of either *clpB* or *clpX* during a 40 min recovery period after incubation at 50°C, we analyzed insoluble FtsZ levels at 20 min time intervals during recovery (Figure 6B). Notably, we observed that in cells deleted for *clpX*, insoluble FtsZ was present immediately after heat treatment and continued to accumulate throughout the recovery period to a greater extent than in wild type or *clpB* deletion cells. These results suggest that ClpXP prevents accumulation of FtsZ aggregates in cells exposed to extreme thermal stress. Since we observed that insoluble FtsZ levels were elevated in Δ*clpB* strains exposed to mild heat shock (Figure S4D), we repeated the recovery time course in *clpX* and *clpB* deletion strains after mild heat shock, 42°C for 30 min, to monitor insoluble FtsZ levels (Figure S4E). We observed that insoluble FtsZ accumulates during the recovery period in *clpB* deletion strains after mild heat shock (Figure S4E).

Finally, if ClpXP is active in cells after severe heat shock, then it should not be a thermolabile protein. To determine if ClpXP remains active after exposure to 50°C *in vitro*, we incubated ClpXP in buffer at 50°C for 1 h, and then measured activity after addition of Gfp-ssrA by monitoring the loss of Gfp-ssrA fluorescence. We observed that ClpXP remained active for unfolding and degradation of Gfp-ssrA after incubation at 50°C for 1 h (Figure S4F). As a control, ClpXP was also incubated in buffer at 30°C for 1 h and then assayed for activity. We observed that ClpXP incubated at 30°C was more active than ClpXP incubated at 50°C, suggesting that a partial loss of activity had occurred at high temperature (Figure S4F). However, this assay was performed in the complete absence of other cellular chaperones or substrates and suggests that some ClpXP likely continues to retain activity after exposure to heat stress, while some may become inactivated.

## Discussion

Here, using both a native and an engineered aggregated substrate, we demonstrate that ClpXP has the operational capacity to disassemble and degrade large aggregates that have ClpX degrons. In this study, FtsZ, a native substrate of ClpXP in *E. coli*, was aggregated *in vitro* by thermal stress, and we further show that FtsZ also aggregates *in vivo* when cells are exposed to high temperature (Figures 2A, 6A). The observation that FtsZ is aggregation prone is in agreement with a prior study reporting the presence of FtsZ in intracellular aggregates of Δ*rpoH* cells incubated at 42°C by mass spectrometry (Tomoyasu et al., 2001). FtsZ aggregates are cleared *in vitro* and *in vivo* by ClpXP, and ClpXP does not require the assistance of additional chaperones (Figures 2E,F, 6A). Moreover, in the absence of ClpP, ClpX also promotes disassembly of FtsZ and Gfp-ssrA aggregates indicating that disassembly can also occur by a proteolysis-independent mechanism, although disaggregation is more efficient in the presence of ClpP. ClpXP-mediated disassembly of Gfp-ssrA aggregates requires ATP in experiments monitoring turbidity (Figure 1E). In addition, the Walker B mutation in ClpX, E185Q, which impairs ATP hydrolysis, also impairs disaggregation of aggGfp-ssrA and, to a lesser extent, aggFtsZ. Aggregate disassembly and resolubilization by ClpX was previously described using the substrate lambda O protein, and here we show disassembly of aggregates and kinetic monitoring using two additional substrates, as well as reactivation of Gfp-ssrA fluorescence (Wawrzynow et al., 1995). Reactivation of Gfp-ssrA is largely dependent on ATP hydrolysis (Figure 3B), since ClpX(E185Q) only weakly promotes reactivation of aggregated Gfp-ssrA (Figure 5C), yet ClpX(E185Q) is capable of stable interactions with substrates in the presence of ATP, although they are not unfolded (Hersch et al., 2005; Camberg et al., 2014). It is unlikely that there are soluble, unfolded Gfp-ssrA monomers in solution after heating, since we did not detect them by DLS and it has been demonstrated that soluble, unfolded Gfp rapidly refolds, in 20–30 s, by a spontaneous reaction that does not require chaperones (Figure 1C; Makino et al., 1997; Tsien, 1998; Zietkiewicz et al., 2004). Therefore, it is likely that large aggregates contain loosely associated unfolded proteins, which can be removed and reactivated by ClpX and, in the case of Gfp-ssrA, allowed to spontaneously refold. As expected, recognition by ClpX is highly specific, as Gfp without an ssrA-tag is not reactivated (Figure 4A).

We also detected partial disaggregation of aggFtsZ by ClpX, but not by ClpX(E185Q) (Figure 5B). Aggregation of FtsZ is induced at 65°C, but the aggregates formed by FtsZ are smaller than those formed by Gfp-ssrA (30 and 600 nm, respectively; Figures 1C, 2D). FtsZ aggregates likely contain 8–10 monomers, based on the average size of a folded FtsZ monomer, which is ~40 Å in diameter (Figure 2D; Oliva et al., 2004). In contrast, Gfp aggregates in this study likely contain more than 120 subunits, based on an average size of a folded Gfp monomer, which is ~50 Å across the long axis (van Thor et al., 2005). The small size of the FtsZ aggregate may allow it to be more susceptible to disassembly by ClpX than a larger aggregate.

**Figure 7:**
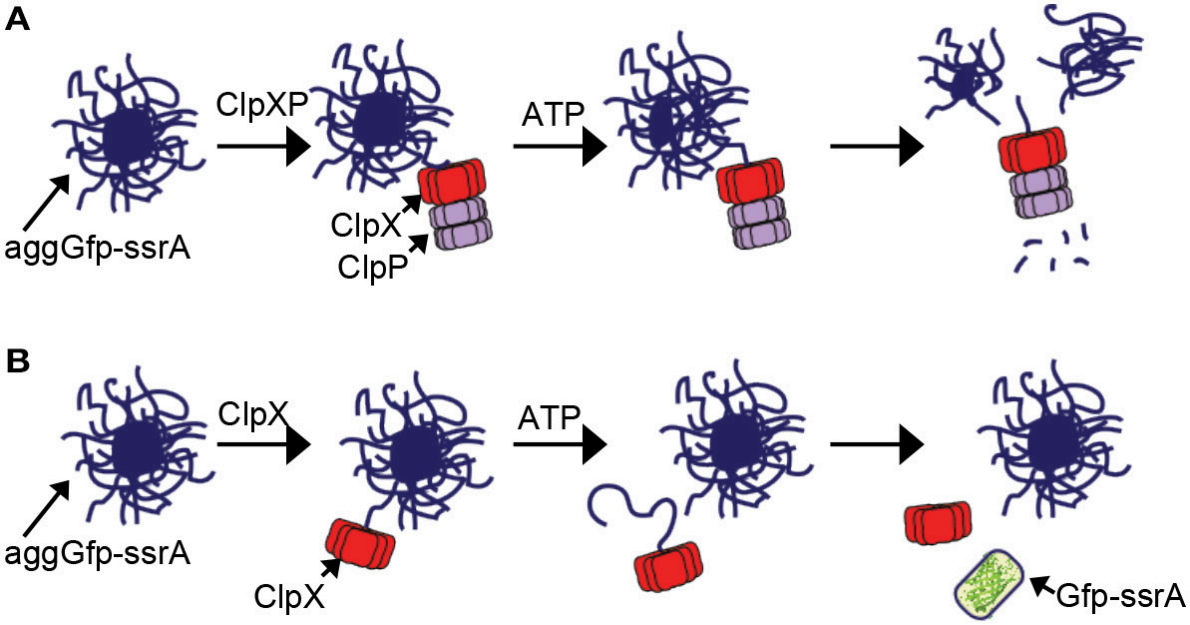
**Model of aggregate disassembly (A)** ClpXP binds to aggregated substrates bearing a ClpX-recognition motif. ClpXP unfolds and degrades protomers from within the aggregate, leading to fragmentation and disassembly in an ATP-dependent manner. **(B)** ClpX binds to aggregates that contain unfolded proteins bearing a ClpX-recognition motif. Unfolded proteins loosely associated with the aggregate surface are reactivated by ClpX through a direct protein interaction that requires ATP-dependent unfolding.

In the model for disassembly of aggregates by ClpXP, ClpX binds to exposed recognition tags on the surface of the aggregate and promotes removal, unfolding and degradation of protomers from within the aggregate (Figure 7A). Removal of protomers eventually leads to destabilization and fragmentation of the aggregate as well as degradation (Figures 1F, 2F). Although this process does not require ClpP, it occurs more robustly when ClpP is present than when ClpP is omitted (Figures 1E, 2E). For aggregated substrate reactivation, ClpX likely engages unfolded protomers from the aggregate, which may be internal or loosely bound to the exterior of the aggregate, unfolds and release them. For small aggregates, this activity may be sufficient to lead to fragmentation and capable of promoting reactivation of substrates such as Gfp-ssrA (Figure 7B).

Finally, we observed large increases in insoluble FtsZ when cells were exposed to two different temperatures, 50°C, which represents extreme heat shock, and 42°C, which represents a mild heat shock (Figures 6A,B, Figure S4D). At 42°C, deletion of *clpB* was associated with a large accumulation of insoluble FtsZ, suggesting that under mild heat stress, ClpB is the major factor that ensures FtsZ solubility (Figures S4D,E). However, we observed a remarkably different result after heat shock at 50°C and throughout the recovery period. Specifically, in a *clpX* deletion strain, large amounts of insoluble FtsZ accumulate during the recovery period to a greater extent than in a *clpB* deletion strain (Figures 6A,B). It is unknown if ClpXP and ClpB are processing FtsZ aggregates directly *in vivo*, because we did not observe a reduction of aggregated FtsZ during the recovery period for any strain. FtsZ is typically present at very high levels (5,000–20,000 copies per cell) and is essential for cell division in *E. coli* (Bramhill, 1997). Interestingly, FtsZ also forms linear polymers as part of its normal biological function to promote cell division, and polymers are efficiently recognized, disassembled, and degraded by ClpXP (Figures 2F,G; Camberg et al., 2009, 2014; Viola et al., 2017). Given the diverse conformational plasticity of FtsZ, its use as a model disaggregation and remodeling substrate will be informative for studies of targeting and processing of multisubunit substrates by AAA+ proteins. As with FtsZ, many other ClpXP substrates are detectable in protein aggregates in cells (Flynn et al., 2003; Maisonneuve et al., 2008). Moreover, a previous study showed that ClpXP is important for cell viability under thermal stress conditions in cells depleted of DnaK (Tomoyasu et al., 2001). Given that it is estimated that 2–3% of *E. coli* proteins are ClpXP substrates, ClpXP likely serves as an additional mechanism to manage accumulation of aggregation-prone proteins *in vivo*, particularly under extreme stress conditions (Flynn et al., 2003; Maisonneuve et al., 2008).

## Author contributions

CL, SM, MV, and JLC conceived and designed the experiments and wrote the paper. CL, SM, MV, JC, and JLC performed the experiments and analyzed the data.

## Funding

This work was funded by an Institutional Development Award (IDeA) from the National Institute of General Medical Sciences of the National Institutes of Health (#P20GM103430 to JLC). The funders had no role in study design, data collection and interpretation, or the decision to submit the work for publication.

### Conflict of interest statement

The authors declare that the research was conducted in the absence of any commercial or financial relationships that could be construed as a potential conflict of interest.

## Supplementary material

Figure S1 **Heat-aggregation of Gfp-ssrA**. The fluorescence emission of aggGfp-ssrA (1.0 μM) (black circles) was monitored as described in Materials and Methods for 90 min.

Figure S2 **Unfolding and degradation of aggregated Gfp-ssrA by ClpXP**. Unfolding and degradation were monitored for aggGfp-ssrA (1.0 μM) alone (black circles) or in the presence of ClpP (0.3 μM) (gold circles), ClpX (0.3 μM) and ClpP (0. μM) (red circles) with ATP (4 mM), where indicated. Fluorescence emission (AU) was monitored as described in Materials and Methods.

Figure S3 **Degradation of FtsZ and FtsZ(ΔC67) by ClpXP**. Degradation was monitored for FtsZ (6 μM) and FtsZ(ΔC67), ClpXP (0.5 μM), ATP (4 mM), GMPCPP (0.5 mM), and a regenerating system where indicated at 23°C for 120 min as described in Materials and Methods, and samples were analyzed by SDS-PAGE and Coomassie stain.

Figure S4 **Insoluble FtsZ in deletion strains after heat-treatment. (A)** Single gene deletion strains (Table 1) were incubated at 50°C for 1 h and recovered as described in Materials and Methods. Cells from deletion strains were collected and insoluble protein extracts were collected as described and analyzed by reducing SDS-PAGE. Immunoblots were performed with antibodies to FtsZ or ClpX as described. **(B)** Total protein present in insoluble cell extracts shown in **(A)** after heat shock at 50°C and recovery was detected by transferring proteins to a nitrocellulose membrane and staining with Ponceau. **(C)** Cell viability for all strains in **(A)** was determined by measuring colony forming units (CFU ml^−1^) of cultures before heating (“pre-HS”), after heat treatment at 50°C for 1 h (“post-HS”), and after 35 min of recovery at 30°C (“post-rec”). **(D)** FtsZ levels were compared in single gene deletion strains after heat shock at 42°C for 30 min and recovery (30°C) as described in Materials and Methods. Cells were collected and insoluble protein extracts were analyzed by immunoblotting with antibodies to FtsZ as described. **(E)** Insoluble FtsZ levels were monitored in wild type, Δ*clpX* and Δ*clpB* deletion strains before heat shock (50°C for 1 h or 42°C for 30 min, where indicated) and during the 30°C recovery period (0, 20, and 40 min). At the indicated times, cells were collected from cultures and insoluble protein extracts were analyzed by immunoblotting with antibodies to FtsZ as described. **(F)** Thermal stability of ClpXP was assayed by incubation of ClpX (0.5 μM) and ClpP (0.7 μM) in phosphate buffered saline supplemented with ATP (4 mM) MgCl_2_ (10 mM), glycerol (15%), Triton X-100 (0.005%), and TCEP (1 mM). Reactions containing ClpXP were added to a preheated quartz cuvette attached to a circulating water bath set to 50 or 30°C, where indicated, and incubated for 1 h. The circulating water bath was rapidly cooled to 30°C, the reactions were supplemented with ATP and regenerating system, Gfp-ssrA (0.2 μM) was added, and fluorescence was monitored with time in the absence (black) or presence of ClpXP, treated at 50°C (red) or 30°C (aqua).
