# Supplementary figures and images for: The Protein Chaperone ClpX Targets Native and Non-native Aggregated Substrates for Remodeling, Disassembly, and Degradation with ClpP
Source: Front Mol Biosci. 2017 May 4;4:26. doi: 10.3389/fmolb.2017.00026 (PMC5415555; doi:10.3389/fmolb.2017.00026)

Figure S1

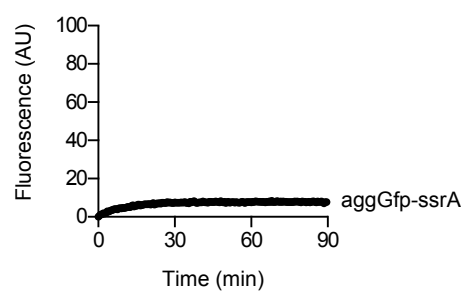

Supplement: Figure S1 — Heat-aggregation of Gfp-ssrA. The fluorescence emission of aggGfp-ssrA (1.0 μM) (black circles) was monitored as described in Materials and Methods for 90 min. [file Image1.PDF]

Figure S2

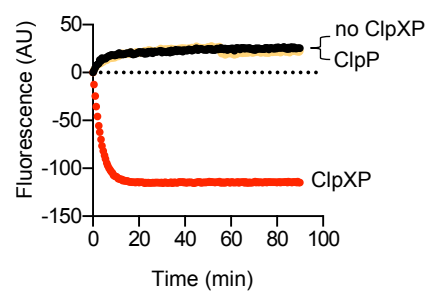

Supplement: Figure S2 — Unfolding and degradation of aggregated Gfp-ssrA by ClpXP. Unfolding and degradation were monitored for aggGfp-ssrA (1.0 μM) alone (black circles) or in the presence of ClpP (0.3 μM) (gold circles), ClpX (0.3 μM) and ClpP (0. μM) (red circles) with ATP (4 mM), where indicated. Fluorescence emission (AU) was monitored as described in Materials and Methods. [file Image2.PDF]

Figure S3

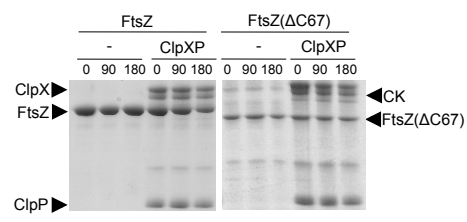

Supplement: Figure S3 — Degradation of FtsZ and FtsZ(ΔC67) by ClpXP. Degradation was monitored for FtsZ (6 μM) and FtsZ(ΔC67), ClpXP (0.5 μM), ATP (4 mM), GMPCPP (0.5 mM), and a regenerating system where indicated at 23°C for 120 min as described in Materials and Methods, and samples were analyzed by SDS-PAGE and Coomassie stain. [file Image3.PDF]

Figure S4

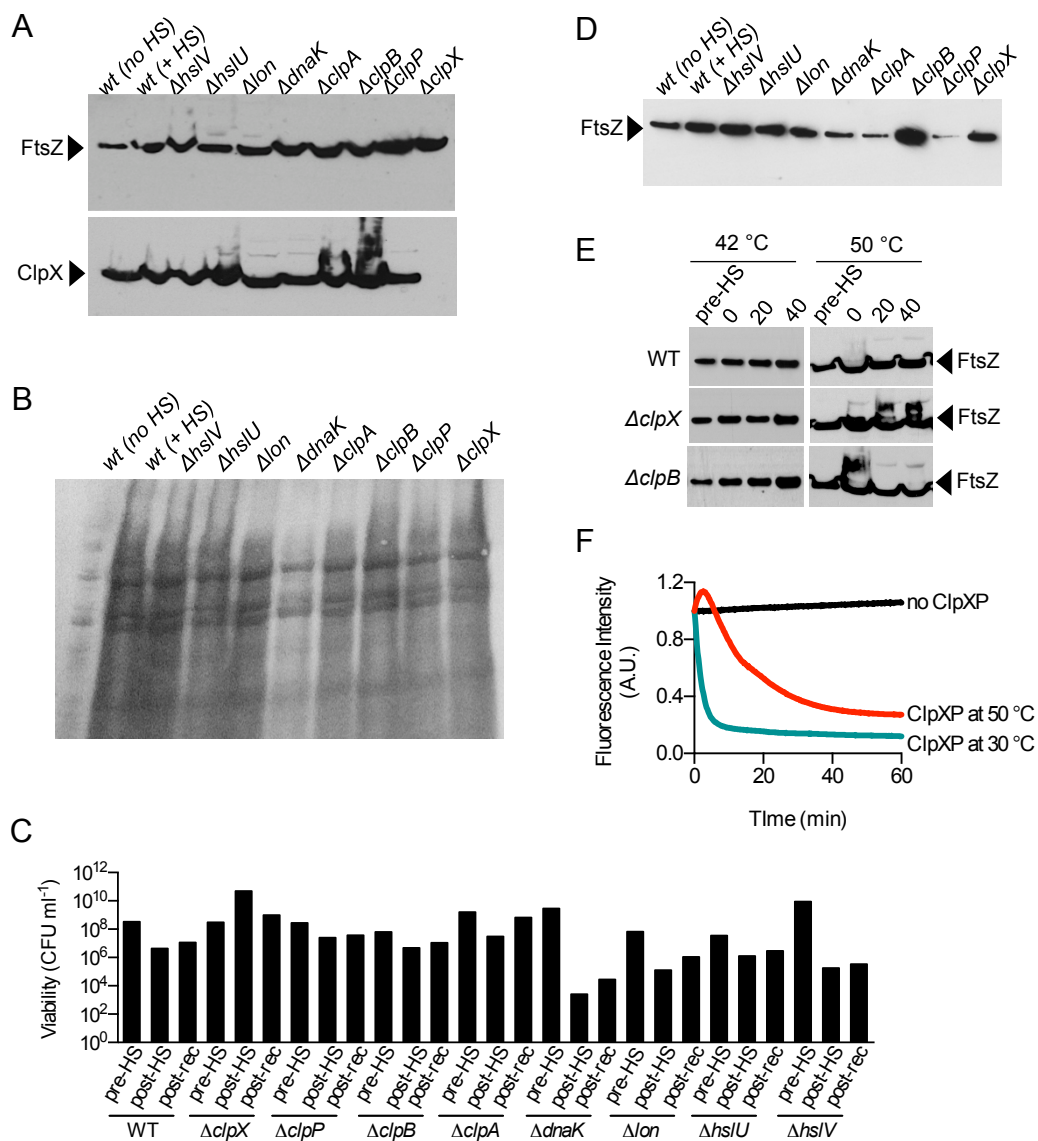

Supplement: Figure S4 — Insoluble FtsZ in deletion strains after heat-treatment. (A) Single gene deletion strains (Table 1) were incubated at 50°C for 1 h and recovered as described in Materials and Methods. Cells from deletion strains were collected and insoluble protein extracts were collected as described and analyzed by reducing SDS-PAGE. Immunoblots were performed with antibodies to FtsZ or ClpX as described. (B) Total protein present in insoluble cell extracts shown in (A) after heat shock at 50°C and recovery was detected by transferring proteins to a nitrocellulose membrane and staining with Ponceau. (C) Cell viability for all strains in (A) was determined by measuring colony forming units (CFU ml−1) of cultures before heating (“pre-HS”), after heat treatment at 50°C for 1 h (“post-HS”), and after 35 min of recovery at 30°C (“post-rec”). (D) FtsZ levels were compared in single gene deletion strains after heat shock at 42°C for 30 min and recovery (30°C) as described in Materials and Methods. Cells were collected and insoluble protein extracts were analyzed by immunoblotting with antibodies to FtsZ as described. (E) Insoluble FtsZ levels were monitored in wild type, ΔclpX and ΔclpB deletion strains before heat shock (50°C for 1 h or 42°C for 30 min, where indicated) and during the 30°C recovery period (0, 20, and 40 min). At the indicated times, cells were collected from cultures and insoluble protein extracts were analyzed by immunoblotting with antibodies to FtsZ as described. (F) Thermal stability of ClpXP was assayed by incubation of ClpX (0.5 μM) and ClpP (0.7 μM) in phosphate buffered saline supplemented with ATP (4 mM) MgCl2 (10 mM), glycerol (15%), Triton X-100 (0.005%), and TCEP (1 mM). Reactions containing ClpXP were added to a preheated quartz cuvette attached to a circulating water bath set to 50 or 30°C, where indicated, and incubated for 1 h. The circulating water bath was rapidly cooled to 30°C, the reactions were supplemented with ATP and regenerating system, Gfp-ssr [file Image4.pdf]
